# Topology-driven activation of the glucagon-like peptide-1 receptor promotes adipose tissue browning

**DOI:** 10.1093/procel/pwag041

**Published:** 2026-06-17

**Authors:** Haiyan Yu, Wenxuan Ma, Guo Yiying, Dongping Zhao, Xiaohan Ye, Kai Chen, Lulu Wang, Jingwu Jian, Wei Zheng, Yu Guo, Dongdong Sun

**Affiliations:** State Key Laboratory of Medicinal Chemical Biology and College of Life Sciences, Academy for Advanced Interdisciplinary Studies, Nankai University, Tianjin 300071, China; Guangzhou Laboratory,Guangzhou,Guangdong 510000,China; State Key Laboratory of Medicinal Chemical Biology and College of Life Sciences, Academy for Advanced Interdisciplinary Studies, Nankai University, Tianjin 300071, China; National Key Laboratory of Intelligent Tracking and Forecasting for Infectious Diseases, School of Statistics and Data Science, Academy for Advanced Interdisciplinary Studies, The Key Laboratory of Pure Mathematics and Combinatorics, Ministry of Education, and Key Laboratory for Medical Data Analysis and Statistical Research of Tianjin, Nankai University, Tianjin 300071, China; State Key Laboratory of Medicinal Chemical Biology and College of Life Sciences, Academy for Advanced Interdisciplinary Studies, Nankai University, Tianjin 300071, China; State Key Laboratory of Medicinal Chemical Biology and College of Life Sciences, Academy for Advanced Interdisciplinary Studies, Nankai University, Tianjin 300071, China; State Key Laboratory of Medicinal Chemical Biology and College of Life Sciences, Academy for Advanced Interdisciplinary Studies, Nankai University, Tianjin 300071, China; State Key Laboratory of Medicinal Chemical Biology and College of Life Sciences, Academy for Advanced Interdisciplinary Studies, Nankai University, Tianjin 300071, China; State Key Laboratory of Medicinal Chemical Biology and College of Life Sciences, Academy for Advanced Interdisciplinary Studies, Nankai University, Tianjin 300071, China; National Key Laboratory of Intelligent Tracking and Forecasting for Infectious Diseases, School of Statistics and Data Science, Academy for Advanced Interdisciplinary Studies, The Key Laboratory of Pure Mathematics and Combinatorics, Ministry of Education, and Key Laboratory for Medical Data Analysis and Statistical Research of Tianjin, Nankai University, Tianjin 300071, China; State Key Laboratory of Medicinal Chemical Biology and College of Life Sciences, Academy for Advanced Interdisciplinary Studies, Nankai University, Tianjin 300071, China; Guangzhou Laboratory,Guangzhou,Guangdong 510000,China; State Key Laboratory of Medicinal Chemical Biology and College of Life Sciences, Academy for Advanced Interdisciplinary Studies, Nankai University, Tianjin 300071, China; Guangzhou Laboratory,Guangzhou,Guangdong 510000,China

**Dear Editor,**

Obesity and type 2 diabetes represent an escalating global health burden, underscoring the need for fundamentally new strategies to modulate metabolic signaling. The glucagon-like peptide-1 receptor (GLP-1R) is a thoroughly validated metabolic target that, over the past decade, has underpinned the development of several highly effective therapeutic modalities (Kim et al., 2024; Xie et al., 2025). Here, we introduce a class of topology-dependent agonists that modulate GLP-1R signaling by imposing defined ­receptor geometries. Through controlled dimerization or oligomerization, these engineered ligands constrain extracellular domain (ECD)–transmembrane domain (TMD) conformations, thereby inducing GLP-1R activation (Moore et al., 2025; Ussher and Drucker, 2023). Importantly, in animal models, a bivalent DARPin (Designed ankyrin repeat protein)-based GLP-1R agonist markedly increased brown adipose tissue (BAT) content and thermogenesis, thereby promoting weight loss via a predominantly appetite-independent mechanism.

Activation of class B G protein-coupled receptors is tightly regulated by allosteric coupling between the ECD and the TMD (Zhang et al., 2017); the ECD also exerts an important influence on receptor conformational states and downstream signaling (Cary et al., 2023). Recent high-resolution structures reveal substantial ECD–TMD conformational rearrangements that underpin agonist efficacy and signaling bias (Kim et al., 2025). These observations support an alternative strategy: rather than solely targeting orthosteric binding sites, imposing defined receptor geometries via controlled dimerization or oligomerization can modulate downstream effector coupling and refine signaling specificity (Harikumar et al., 2012). Notably, engineered surrogate ligands and designed protein scaffolds have demonstrated a key principle in receptor modulation. DARPin-based C2-symmetric mimetics show that precise tuning of inter-monomer angles and distances yields predictable, biased signaling outputs in dimeric receptors (Mohan et al., 2019; Moraga et al., 2015). This framework provides an alternative strategy for modulation of GLP-1R activity.

In this study, we explored a topology-driven activation strategy for GLP-1R by inducing ECD-mediated receptor dimerization with DARPins. By combining DARPin library screening with AI-based structure, we generated bivalent DARPin fusion proteins and evaluated their cAMP signaling and insulinotropic activity in cells, followed by a comprehensive assessment of metabolic efficacy in mouse models (Fig. 1A).

**Figure 1.**
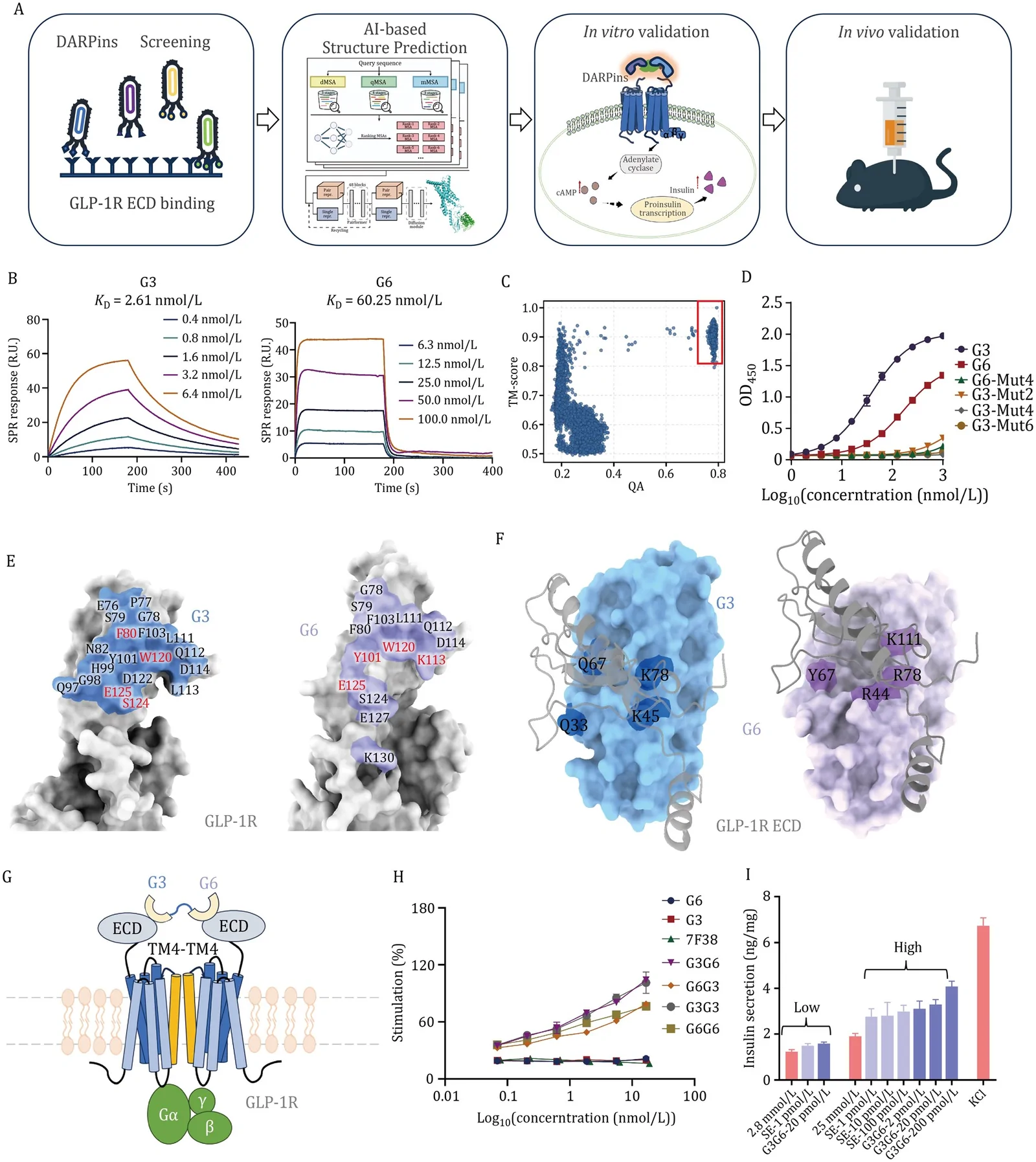
**Identification and functional characterization of engineered DARPins as GLP-1R activators**. (A) Schematic diagram of the experimental design. DARPins, identified by experimental screening combined with AI prediction design, were validated *in vitro* and *in vivo* models. (B) Surface plasmon resonance (SPR) sensorgrams showing the binding affinity of monomeric DARPins. (C) Scatter plot of QA vs. TM-score for all G3-GLP-1R docking models. Each point represents a single model. Models segregate into distinct clusters, with a high-QA/high-TM-score cluster (boxed) representing conformations that converge toward the top-ranked binding mode, reflecting increased structural consistency and confidence. (D) ELISA-based dose–response analysis of DARPin binding to the GLP-1R ECD. Binding signals (OD_450_) were measured across the indicated concentration range and are shown as mean ± SD (*n* = 3). G3 and G6 denote the parental DARPins, while interface mutants include G3-Mut2, G3-Mut4, G3-Mut6 (Q33A, R44A, K45A, Q67A, W77A, K78A), and G6-Mut4. (E) Key binding sites of DARPin G3 and G6 on the GLP-1R ECD. (F) Predicted structures of DARPin-GLP-1R complexes highlighting key interaction residues. G3 (blue), G6 (purple), GLP-1 (red), and GLP-1R (light gray). (G) Structural model of G3G6-induced dimerization of the GLP-1 receptor. (H) cAMP levels induced by G3G6, G3G3, G6G3, and G6G6 were measured and normalized to 100%. Fab7F38, a negative-control Fab that binds the ECD without affecting ligand binding, was included for comparison (Zheng et al., 2024). (I) Glucose-stimulated insulin secretion (GSIS) in MIN6 cells. Cells were incubated under low-glucose (2.8 mM) or high-glucose (16.7 mM) conditions in the presence of G3G6 or semaglutide. G3G6L and SEL denote G3G6-treated and semaglutide-treated cells, respectively, under low-glucose (2.8 mM) conditions. Depolarization with 30 mM KCl was included as a positive control.

We screened the N3C DARPin library (containing three ankyrin-repeat modules) against purified GLP-1R ECD and identified two dominant monomeric binders, G3 and G6 (Fig. S1) (Hennen et al., 2016). To interrogate their binding modes, we predicted GLP-1R/DARPin complex structures with EnsembleFold (Fig. S2) (Wuyun et al., 2026), a deep-learning pipeline built on our CASP15-winning DMFold architecture (Zheng et al., 2024). By integrating DeepMSA2-derived high-quality MSAs with a modified AlphaFold3 architecture, EnsembleFold enables accurate and efficient modeling of protein–protein assemblies. Rather than relying on a single default prediction, EnsembleFold generates and ranks large ensembles of decoy models across multiple MSAs and random seeds. Model quality was evaluated using a QA score that combines the predicted TM-score (pTM) and interface-predicted TM-score (ipTM). G3/GLP-1R models achieved high confidence (top QA = 0.794), whereas G6 models scored lower, consistent with a weaker or more dynamic interaction. Surface plasmon resonance analysis revealed that G3 binds to the GLP-1R ECD with a *K*_D_ of 2.61 nM (*k*_on_  = 4.76 × 10^6^ M^−1^ s^−1^, *k*_off_  = 1.24 × 10^−2^ s^−1^), while G6 exhibited a weaker affinity (*K*_D_  = 60.25 nM; *k*_on_  = 2.34 × 10^6^ M^−1^ s^−1^, *k*_off_  = 0.141 s^−1^), and pre-saturation with G3 blocked subsequent G6 binding, indicating partially overlapping ECD interfaces (Figs. 1B and S3).

Focusing on the high-confidence G3 ensemble, we quantified structural convergence by TM-score and selected 815 models (QA range 0.758–0.794) for interface mapping. Interface residues were defined as DARPin heavy atoms within 5 Å of any GLP-1R heavy atom across the ensemble, enabling construction of a residue-level binding probability map (Figs. 1C and S4) in which high-frequency contacts denote robust determinants. Experimental competition data were used to refine G6 models, which occupy a partially overlapping but noncanonical ECD surface relative to G3 (Table S1). Notably, both DARPins engage sites spatially distinct from the canonical peptide agonist pocket, revealing a previously unrecognized epitope on the GLP-1R ECD (Table S2). To validate the AI-predicted interface, we performed alanine scanning mutagenesis on the DARPin binders. G3-Mut2 (K45A, K78A) introduces substitutions at the closest predicted GLP-1R contact binding residues and results in a pronounced loss of binding across the concentration range. In contrast, more extensive mutations, including G3-Mut4 (Q33A, K45A, Q67A, K78A), G3-Mut6, and G6-Mut4 (R44A, Y67A, R78A, K111A), abolish detectable binding throughout the tested concentrations (Fig. 1D). Key interface residues and the corresponding DARPin-GLP-1R binding architectures further support this interaction mode (Fig. 1E and 1F). Collectively, these results demonstrate that AI-based ensemble structure prediction, when integrated with biophysical constraints and experimental validation, can reliably identify noncanonical GLP-1R binding interfaces and elucidate the structural basis of engineered DARPin modulators (Fig. 1G).

Based on these binding data, we engineered a series of multivalent DARPin constructs by linking different DARPin molecules with a (GGGGS)_2_ linker (Fig. S5). These constructs were then evaluated for their ability to activate GLP-1R-mediated Gαs/cAMP signaling. In CHO-K1 cells stably expressing human GLP-1R and a CRE-driven luciferase reporter, monomeric G3 or G6 alone failed to elevate cAMP above baseline, indicating that single-site ECD binding is insufficient to trigger Gαs signaling (Mohan et al., 2019). In contrast, flexible bivalent constructs (G3G3, G6G6, G3G6, G6G3) elicited robust cAMP responses in the absence of exogenous GLP-1, with G3G6 achieving ∼103.7% of the response induced by the positive control semaglutide (SE) (Figs. 1H and S6). The inactivity of single-site binders, together with the clear dependence on bivalent architecture and linker flexibility, supports a model in which bivalent DARPins enforce ECD-mediated GLP-1R dimerization that is sufficient to activate downstream Gαs/cAMP signaling, indicating a topology-dependent mechanism rather than nonspecific receptor clustering.

Next, we evaluated the effects of the bivalent DARPin G3G6 on insulin secretion using MIN6 mouse pancreatic β-cells. Under high-glucose conditions (16.7 mM), both SE and G3G6 significantly enhanced insulin secretion (Fig. 1I). In particular, 200 pM G3G6 induced a 1.1-fold increase in insulin release compared to high-glucose controls (*P* = 0.0001). Under low-glucose conditions (G3G6, SE), neither agent altered basal insulin secretion, confirming glucose-dependent activity. As a positive control, 30 mM KCl depolarization caused a 2.5-fold increase in insulin secretion (*P* < 0.0001). These data demonstrate that G3G6 potentiates glucose-stimulated insulin secretion in β-cells, recapitulating an incretin-like effect.

Based on the potent *in vitro* signaling and insulinotropic activity, we next evaluated the metabolic pharmacology of the bivalent DARPin G3G6 in a high-fat diet (HFD)-induced obesity model in C57BL/6J mice. The results revealed a fundamental mechanistic divergence from conventional GLP-1R agonism. Following 16 days of intraperitoneal administration, the PBS control group retained a marked obesity phenotype, whereas both SE and G3G6 treatments elicited weight loss (Fig. S7). Endpoint measurements confirmed a 5.7% body weight reduction in the G3G6 group compared to PBS controls (Fig. 2A). Crucially, monitoring of daily food intake revealed a clear difference between treatments: SE consistently reduced food consumption (8.4% overall reduction, with peak suppression of 13.8% at Day 12), while G3G6-treated mice showed food intake levels largely comparable to the PBS group (2.3% overall difference, *P* = 0.81), despite a transient reduction observed at Day 12 (Fig. S8). This distinct profile suggests that G3G6 drives weight loss predominantly through an appetite-independent mechanism, likely involving enhanced energy expenditure.

**Figure 2.**
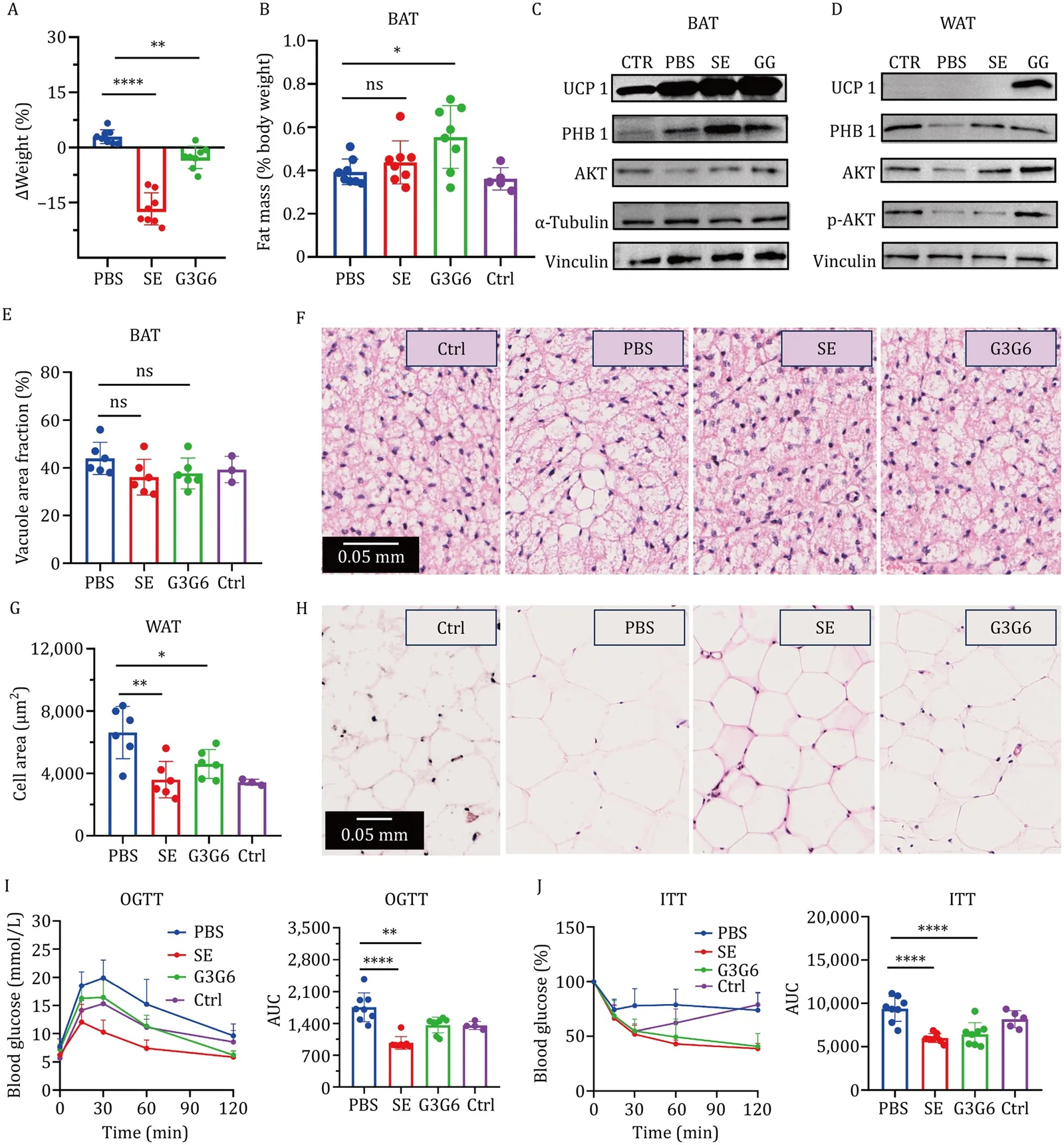
***In vivo* efficacy of the bivalent DARPin G3G6 in a high-fat diet mouse model**. (A) Body weight trajectories and endpoint Δweight. G3G6 vs. PBS, *P* = 0.0043; semaglutide vs. PBS, *P* < 0.0001. (B) Interscapular BAT mass (% body weight). G3G6 vs. PBS, *P* = 0.0103. (C) Representative Western blot analysis of thermogenic and metabolic signaling proteins in brown adipose tissue (BAT), including UCP1 (uncoupling protein 1), PHB1 (prohibitin 1), and total AKT (protein kinase B). (D) Representative Western blot of key proteins, including UCP1, PHB1, total AKT, and phosphorylated AKT (pAKT) in WAT. (E) Quantification of vacuolar area in BAT. (F) Representative H&E staining of WAT. (G) Quantification of mean adipocyte area in WAT. G3G6 vs. PBS, *P* = 0.0297; semaglutide vs. PBS, *P* = 0.0014. (H) Representative H&E staining of BAT. (I) OGTT curves and AUC analysis in normal, PBS, semaglutide, and G3G6 groups. G3G6 vs. PBS, *P* = 0.0033; semaglutide vs. PBS, *P* < 0.0001. (J) ITT curves and AUC analysis. G3G6 vs. PBS, *P* < 0.0001; semaglutide vs PBS, *P* < 0.0001. Data are presented as mean ± SEM. Statistical significance is indicated as follows: *P < 0.05; **P < 0.01; ***P < 0.001; ****P < 0.0001; ns, not significant.

To unravel the basis of this appetite-independent effect, we performed a detailed body composition analysis. Although muscle mass was comparable across groups, both treatments significantly reduced white adipose tissue (WAT) mass proportion, with a preferential loss of visceral fat depots (Fig. S9). The most notable difference emerged in BAT. G3G6 treatment selectively increased the BAT mass proportion by 41% compared to PBS controls (*P* = 0.01), whereas SE induced only a non-significant 11% increase (*P* = 0.73) (Fig. 2B).

To validate the functional integrity of the expanded BAT and explore the molecular signature of adipose remodeling, we quantified key thermogenic regulators (Fig. 2C and 2D). G3G6 markedly upregulated canonical thermogenic markers UCP1 in BAT (Fig. S10A–C) (Yao et al., 2017). A concurrent induction of UCP1 in WAT is indicative of browning, while elevated pAKT levels point to recruitment of PI3K-AKT signaling in adipose depots, which may contribute to improved peripheral insulin sensitivity (Fig. S10D–G). These molecular changes confirm that the G3G6-induced BAT expansion corresponds to functionally hyperactive, thermogenic tissue. Histological analysis corroborated these findings. G3G6 exerted a pronounced protective effect on BAT morphology, effectively preserving the healthy, multilocular architecture essential for thermogenesis, in contrast to the unilocular lipid accumulation and “whitening” observed in PBS controls (Fig. 2E and 2F). In WAT, adipocytes from the G3G6 group showed a trend toward reduced size (Fig. 2G and 2H). Collectively, we conclude that G3G6 drives weight loss through a mechanistically distinct, primarily appetite-independent pathway. This is achieved primarily via the preservation of BAT structural integrity and a potent amplification of its intrinsic thermogenic capacity, starkly contrasting with the anorectic effect of SE and suggesting a novel, topology-dependent engagement of energy expenditure pathways.

Finally, we evaluated whether these tissue-level changes translated into systemic metabolic improvements. In glucose tolerance tests (OGTT), G3G6-treated mice exhibited a significant 22.3% reduction in the area under the curve (AUC) compared to PBS controls (*P* < 0.01). The glucose excursion curves closely resembled those of normal controls, although SE produced a more pronounced suppression (Fig. 2I). In insulin tolerance tests (ITT), G3G6 improved insulin sensitivity by reducing the AUC by 31.2% relative to PBS (*P* < 0.0001), an effect comparable to SE’s 36.0% reduction (Fig. 2J). Beyond glycemic control, G3G6 exerted a broad regulatory effect on lipid metabolism, effectively lowering triglycerides (−27.5%, *P* = 0.297) and low-density lipoprotein (LDL) cholesterol (−27.0%, *P* < 0.0001), accompanied by reductions in high-density lipoprotein (HDL) cholesterol (−24.4%, *P* = 0.031) and total cholesterol (−14.7%, *P* = 0.127; not statistically significant) (Fig. S11A–D). Furthermore, hepatic function assays indicated modest hepatoprotective potential, with serum alanine aminotransferase (ALT) and aspartate aminotransferase (AST) activities reduced by 25.4% and 27.4%, respectively, compared to PBS controls (Fig. S11E and S11F). In summary, G3G6 not only recapitulates the core glucoregulatory benefits of conventional GLP-1R agonism by improving glucose tolerance and insulin sensitivity but also extends the therapeutic profile by conferring distinct advantages in lipid metabolism, primarily through significant reductions in triglycerides and LDL cholesterol, along with indications of improved hepatic health.

From initial functional screening to comprehensive metabolic phenotyping, our study systematically elucidates the critical role of enforced GLP-1R dimerization in the fine-tuning of receptor signal transduction. Building on prior findings that GLP-1R can form homodimers and that its oligomeric state influences signaling bias (Wu et al., 2020), we discovered that a bivalent DARPin G3G6 binds to a noncanonical epitope on the ECD of GLP-1R and robustly activates the Gαs/cAMP signaling pathway independently of the endogenous ligand. This mode of action is fundamentally distinct from that of traditional orthosteric agonists. At a broader level, this work not only establishes a new class of GLP-1R modulators but also provides important insights into the regulatory mechanisms of Class B GPCR signaling, highlighting that receptor dimerization itself can serve as a crucial molecular switch for signal activation.

Importantly, the mechanism of weight loss induced by G3G6 *in vivo* is fundamentally distinct from that of classical GLP-1R agonists. Unlike SE, which achieves weight reduction primarily by suppressing appetite and reducing food intake, G3G6 markedly induces the expansion of BAT. This expansion is characterized by the persistence of multilocular lipid droplet morphology and robust upregulation of UCP1, indicating effective activation of a thermogenic program driven by the cAMP-PKA-CREB axis. By selectively promoting BAT expansion and enhancing thermogenesis, G3G6 mediates weight loss without affecting feeding behavior. In summary, we establish the bivalent DARPin G3G6 as a topology-driven modulator that enforces GLP-1R dimerization to activate BAT thermogenesis and weight loss through a pathway that is largely independent of appetite suppression. These findings offer a novel conceptual framework and therapeutic strategy for the treatment of metabolic diseases.

## Supplementary data

Supplementary data are available at *Protein & Cell* online https://doi.org/10.1093/procel/pwag041

## Footnotes

We sincerely thank Prof. Zhang Hongkai’s research group at Nankai University for providing CHO-K1 cells.

ALT, alanine aminotransferase; AST, aspartate aminotransferase; AUC, area under the curve; BAT, brown adipose tissue; DARPin, Designed ankyrin repeat protein; ECD, extracellular domain; GLP-1R, glucagon-like peptide-1 receptor; HDL, high-density lipoprotein; HFD, high-fat diet; ipTM, interface-predicted TM-score; ITT, insulin tolerance tests; LDL, low-density lipoprotein; OGTT, glucose tolerance tests; pTM, predicted TM-score; SE, semaglutide; TMD, transmembrane domain; WAT, white adipose tissue.

H.Y., W.M., Y.Y.G., and D.Z. contributed equally to this work. H.Y. conceived and led the study, designed the research strategy, performed and coordinated the majority of experiments throughout the project, analyzed data, and drafted the manuscript. W.M. carried out the *in vivo* (animal) experiments and analyzed the related data; Y.Y.G. performed structural modeling and computational analyses; and D.Z. led DARPin selection and binding validation experiments. X.Y. contributed to *in vitro* experiments, data collection, and figure preparation. K.C. and L.W. assisted with methodology development, sample processing, and technical support. J.J. assisted with the development and validation of the preliminary obesity model used to design the *in vivo* experiments. Project supervision, funding acquisition, and critical manuscript revision were provided by D.S., Y.G., and W.Z. All authors discussed the results, contributed to revision of the manuscript, and approved the final version.

The authors declare that they have no competing financial or non-financial interests related to this work. The research was conducted in the absence of any commercial or financial relationships that could be construed as a potential conflict of interest.

This work was supported by the National Natural Science Foundation of China (12426303 to W.Z.), the Fundamental Research Funds for the Central Universities (030–63253192 to Y.G., 054–63253109 to W.Z.), the Tianjin Science and Technology Program (25ZXZSSS00460 to W.Z.), Tengfei initiative by the National Key Laboratory of Intelligent Tracking and Forecasting for Infectious Diseases (2025NITFID510 and 2026NITFID510 to W.Z.), and the Fundamental and Interdisciplinary Disciplines Breakthrough Plan of the Ministry of Education of China (JYB2025XDXM503 to Y.G.).

All data supporting the conclusions of this study are provided within the article and its supplementary information (including source data files, Tables S1 and S2, and Figs. S1–11).

This study did not generate new custom code. All analyses were performed using publicly available software as described in the Methods section.

This study was approved by the Ethics Committee of the State Key Laboratory of Medicinal Chemical Biology at Nankai University (Ethics Approval Number: 2025-SYDWLL-000826). All sample collection and laboratory procedures were conducted in accordance with the approved protocols and relevant guidelines.

In this study, deep learning-based tools, EnsembleFold (built on DMFold and AlphaFold3 architectures), were used for protein complex structure prediction and model quality assessment (e.g., pTM and ipTM scores). These AI-assisted predictions were benchmarked against experimental data (surface plasmon resonance) and interpreted solely by the authors.

## Supplementary Material

pwag041_Supplementary_Data
