# Supplementary material for: Topology-driven activation of the glucagon-like peptide-1 receptor promotes adipose tissue browning
Source: Protein Cell. 2026 Jun 17;17(11):1066–71. doi: 10.1093/procel/pwag041 (PMC13721117; doi:10.1093/procel/pwag041)
Supplement: Supplementary file 1 [file pwag041_supplementary_data.docx]

**Supplementary information for**

**Topology-Driven Activation of the Glucagon-Like Peptide-1 Receptor Promotes Adipose Tissue Browning**

Haiyan Yu^1,2†^, Wenxuan Ma^1†^, Yiying Guo^3†^, Dongping Zhao^1†^, Xiaohan Ye^1^, Kai Chen^1^, Lulu Wang^1^, Jingwu Jian^1^, Wei Zheng^3#^, Yu Guo^1,2#^, Dongdong Sun^1,2#^

^1^State Key Laboratory of Medicinal Chemical Biology and College of Life Sciences, AAIS, Nankai University, Tianjin 300071, China;

^2^Guangzhou Laboratory, Guangzhou, Guangdong, People’s Republic of China.

^3^NITFID, School of Statistics and Data Science, AAIS, LPMC, and KLMDASR, Nankai University, Tianjin, 300071, China.

^†^ These authors contributed equally to this work.

^#^Correspondence: [16622892369@163.com](mailto:16622892369@163.com) (D. S), guoyu@nankai.edu.cn (Y. G), [jlspzw@nankai.edu.cn](mailto:jlspzw@nankai.edu.cn) (W.Z).

Fig. S1 to Fig. S11

Tab. S1 to Tab. S2

**Methods**

**Expression and purification of GLP-1R ECD and GLP-1R-targeting DARPins**

Human GLP-1R ECD (residues 1-148, UniProt P43220) with a C-terminal 6 $\times$ His tag was cloned into pFUSE (InvivoGen) and transiently expressed in suspension HEK293F cells (1 $\times$ 10^6^ cells/mL). Cells were transfected with plasmid DNA using polyethyleneimine (PEI) and cultured at 37°C, 5% CO_2_ for 5 days in the presence of 5 μM kifunensine (MedChemExpress#109944-15-2) to enrich high-mannose N-glycans. The culture supernatant was clarified by centrifugation (4,000 g, 20 min, 4°C). GLP-1R ECD was captured from the supernatant using Ni-NTA agarose (GE Healthcare) pre-equilibrated in PBS (pH 7.4), washed with PBS containing 50 mM imidazole, and eluted with 500 mM imidazole. The eluate was concentrated (10 kDa MWCO, Millipore) and buffer-exchanged into PBS. The protein was further purified by size-exclusion chromatography (Superdex 75 Increase 10/300 GL, Cytiva) at 4°C; the monodisperse peak was collected. The pooled protein was incubated with endoglycosidase H (Endo Hf) at 1:1000 w/w overnight at 4°C to remove N-glycans. Final purity ($>$95%) was confirmed by Coomassie-stained 15% SDS-PAGE, and concentration was measured by NanoDrop using the theoretical extinction coefficient.

High-affinity DARPins G3 and G6 were fused head-to-tail via a linker to generate the tandem construct. Monomeric G3, G6, and homodimeric G3G3, G6G6, G3G6, G6G3, constructs (each with a C-terminal 6$\times$ His tag) were cloned into pET-22b ($+$). The plasmids were transformed into *E*. *coli* BL21(DE3) and selected on LB agar with 100 μg/mL ampicillin. A single colony was used to inoculate 10 mL LB (100 μg/mL ampicillin) and grown at 37°C for 10 h, then diluted 1:100 into 1 L LB. Cultures were grown at 37°C to OD_600_ $\approx$ 0.6, then cooled to 16°C and protein expression was induced with 300 μM IPTG for 20 h. Cells were harvested and lysed by high-pressure homogenization (APV-1000, 1000 bar, 10 min) in cold PBS (pH 7.4). The lysate was clarified by centrifugation (18,000 g, 45 min, 4°C). His-tagged DARPins were purified from the supernatant by Ni-NTA affinity chromatography (Qiagen). The eluates were polished by size-exclusion chromatography on a Superdex 75 10/300 GL column (Cytiva) at 4°C to isolate monomeric protein.

**Structure prediction of GLP-1R in complex with DARPins**

The tertiary structures of GLP-1R in complex with the DARPins G3 and G6 were predicted utilizing EnsembleFold, a newly developed biomolecular structure prediction framework built on our CASP15-winning algorithm DMFold. By integrating DeepMSA2 for high-quality multiple sequence alignment (MSA) construction with a modified AlphaFold3 prediction engine, EnsembleFold enables accurate and efficient modeling of protein-ptotein complexes.

The first step of EnsembleFold involves MSA generation using DeepMSA2 to improve protein structure prediction. Starting from the input sequences, the DeepMSA2-Monomer pipeline performs homology searches against a comprehensive collection of genomic and metagenomic databases comprising over 40 billion sequences through three parallel blocks (dMSA, qMSA, and mMSA) to achieve sufficient alignment depth. For the G3/G6–GLP-1R complexes, the top $M$ MSAs from each of the $N$ distinct chains (G3 or G6, and GLP-1R) are paired to form $M^{N}$ hybrid alignments, with $M$ adjusted to balance computational cost. Sequence linking is then performed based on shared UniProt species, with sequences ordered by identity to the query and gaps filled in for any missing chains. Finally, the top 25 multimeric MSAs are selected for G3/G6–GLP-1R complexes based on a combined score incorporating MSA depth and predicted Local Distance Difference Test (pLDDT) scores.

In the second step, structural models were generated powered by a modified AlphaFold3 architecture. The MSAs generated in the first step are fed directly into this modeling engine in place of AlphaFold3 default sequence alignments. Key innovations to the AlphaFold3 modeling engine include (i) using templates or not, (ii) adjusting the dropout rate, (iii) generating a higher number of decoys than the default setting (five models), (iv) applying or omitting the early stop strategy, and (v) increasing or decreasing the number of recycling iterations. For both G3-GLP-1R and G6-GLP-1R complex, ten recycling iterations were employed and 400 models were generated per MSA using 80 random seeds across 25 MSAs, yielding a total of 10,000 models per complex. Final model ranking was performed using the quality assessment (QA) score, which is a linear combination of the predicted TM-score (pTM) and the interface-predicted TM-score (ipTM). A higher QA score indicates greater confidence in the predicted structure and its intermolecular interface.

$$QA=0.8\times iptm+0.2\times ptm$$

**Surface plasmon resonance (SPR) analysis**

Binding kinetics were measured on a Biacore T200 (Cytiva) at 25°C. The GLP-1R ECD was immobilized on a CM5 sensor chip by standard amine coupling: the surface was activated with 0.4 M EDC/0.1 M NHS, and 10 μg/mL GLP-1R ECD in 10 mM sodium acetate (pH 5.0) was injected at 10 μL/min until $\sim$357 response units (RU) were bound. The surface was quenched with 1 M ethanolamine (pH 8.5). A reference flow cell was activated and quenched in parallel without protein. G3 and G6 were diluted in HBS-EP running buffer (10 mM HEPES, 150 mM NaCl, 3 mM EDTA, 0.005% P20, pH 7.4) to final concentrations of 0.39-12.5 nM and 6.25-100 nM, respectively, using two-fold serial dilutions. Each analyte was injected at 30 μL/min, with a 120 s association phase and a 300 s dissociation phase. The surface was regenerated with 10 mM glycine (pH 1.5) for 30 s between injections. Reference-subtracted sensorgrams were globally fitted to a 1:1 Langmuir binding model to derive the association (k_on_) and dissociation (k_off_) rate constants and equilibrium KD (Biacore T200 Evaluation Software v3.2). Data from duplicate experiments were analyzed using a two-state reaction model (BIAevaluation 2.0.3, GE Healthcare).

**Structural similarity assessment**

The template modeling score (TM-score) was used to quantitatively assess the structural similarity between each predicted G3-GLP-1R model and the top-ranked structure in this work.

$$TM-score=max[\frac{1}{N}\sum_{i=1}^{L} \frac{1}{1+{(\frac{d_{i}}{d_{0}})}^{2}}],$$

where $N$ represents the length of the top-ranked structure, $L$ denotes the number of aligned residues, $d_{i}$ is the Euclidean distance between the $i$-th pair of aligned residues, and $d_{0}=1.24\times\sqrt[3]{N-15}-1.8$ is a distance scaling factor. The maximum is taken over all possible structural superpositions. The TM-score ranges from 0 to 1, with higher values indicating a closer similarity to the top-ranked topology.

**Structural prediction and orientation design of DARPins**

Computational modeling of DARPin geometry was performed using homology templates and the Rosetta suite to ensure that fusion constructs could span the distance between two GLP-1R ECDs in the cell surface dimer. The G3 and G6 binders were modeled onto the GLP-1R ECD structure (PDB: 5VAI) to predict their binding epitopes and approximate orientation. The (GGGGS)_2_ linker length was chosen to allow sufficient flexibility while maintaining a defined dimeric geometry.

**cAMP accumulation assay**

Intracellular cAMP levels were measured using a CRE-driven luciferase reporter in GLP-1R-expressing CHO cells. Cells were plated in 96-well plates (1 $\times$ 10^5^ cells/well) and incubated overnight. DARPin fusion proteins and semaglutide were prepared in complete medium at initial concentrations of 16 μM and 720 nM, respectively, and serially diluted. After replacing the medium, cells were treated with ligands (50 μL per well) and incubated for 6 h at 37°C. Following incubation, 100 μL of ONE-Glo™ luciferase reagent (Promega) was added to each well, plates were shaken in the dark at 37°C for 10 min, and luminescence was measured on a Spark multimode reader (Tecan). Luminescence was normalized to vehicle control.

**Glucose-stimulated insulin secretion (GSIS)**

MIN6 mouse pancreatic β-cells were used to assess insulin secretion. Cells were plated in 24-well plates and incubated overnight in DMEM medium with 10% FBS. For GSIS, cells were pre-incubated for 2 h in Krebs-Ringer bicarbonate buffer with 2.8 mM glucose, then incubated for 1 h with either low or high glucose in the presence or absence of test compounds (semaglutide and G3G6). After incubation, supernatants were collected and insulin was measured by ELISA (Mercodia) and normalized to total cellular protein (BCA assay). As a positive control, cells were depolarized with 30 mM KCl for 1 h. Insulin secretion data are presented as fold-change over baseline (high glucose alone).

**Animal studies**

All animal experiments were conducted at the Laboratory Animal Center of Nankai University under the ethics approval number 2025-SYDWLL-000826. Male C57BL/6J mice (age 4 weeks) were fed a high-fat diet (60% kcal from fat) for 12 weeks to induce obesity. Mice were then randomized into three groups (n $=$ 8 per group) with similar mean body weight. For treatment, semaglutide was administered at 0.06 mg/kg as a positive control. This dose was selected based on published mouse studies demonstrating pharmacological activity of semaglutide in the range of approximately 0.003-0.1 mg/kg, and the chosen 0.06 mg/kg falls within a well‑established, physiologically relevant exposure window for GLP‑1 receptor agonists(Chuong et al., 2023; Piątkowska-Chmiel et al., 2025). G3G6 was administered at 10 mg/kg, a dose consistent with prior in vivo studies of DARPin-based biologics, including the trispecific DARPin ensovibep (10 mg/kg optimal dose(Rothenberger et al., 2022) ), other DARPin therapeutics active at mg/kg levels in mice (Zeng et al., 2022), and clinical DARPin candidates with tolerated doses of 8-12 mg/kg in humans(Baird et al., 2021).

Body weight and food intake were monitored every six days. At study end, mice were fasted for 6 h and subjected to glucose tolerance tests (2 g/kg oral glucose) or insulin tolerance tests (0.75 U/kg intraperitoneal insulin) with glucose measured at indicated time points. Mice were then sacrificed by CO_2_ asphyxiation. Tissues (adipose depots, muscle, liver) were dissected, weighed, fixed in 4% paraformaldehyde, and processed for histology. Blood samples were collected for serum insulin and lipid measurements (ELISA and colorimetric assays).

**WB and Tissue sections**

Epididymal WAT (100 mg) and interscapular BAT (100 mg) were homogenized on ice in RIPA buffer (with 1% protease inhibitor cocktail and PhosSTOP). Lysates were cleared (12,000 g, 15 min, 4°C) and protein concentrations determined by BCA assay (Pierce). Equal amounts of protein (30 μg for eWAT; 15 μg for BAT) were separated by 10% SDS-PAGE (stacking at 80 V; resolving at 120 V) and transferred to PVDF membranes (0.22 μm, Millipore) by wet transfer (100 V, 60 min). Membranes were blocked in TBST (20 mM Tris-HCl, pH 7.6; 137 mM NaCl; 0.1% Tween-20) with 5% nonfat milk for 1 h at room temperature. Membranes were then incubated overnight at 4°C with primary antibodies: anti-UCP1 (rabbit, Abcam ab209483, 1:1,000), anti-phospho-AKT (AKT1 pS473 $+$ AKT2 pS474 $+$ AKT3 pS472, rabbit, Abcam ab192623, 1:1,000), anti-total AKT (rabbit, Abcam ab185633, 1:1,000), anti-PHB1 (rabbit, Selleck f2433, 1:10,000), anti-Vinculin (rabbit, Abways CY5164, 1:2,000) and anti-α-tubulin (mouse, Selleck F0063, 1:10,000). After three washes in TBST, blots were incubated for 1 h at room temperature with HRP-conjugated secondary antibodies (anti-rabbit, Invitrogen #31460, 1:10,000). Immunoreactive bands were developed using ECL Prime (Cytiva) and imaged on a ChemiDoc MP system (Bio-Rad). Band intensities were quantified with Image Lab (Bio-Rad) and normalized to α-tubulin. For total AKT detection, membranes were pretreated with 50 mM NaOH (15 min) before probing with phospho-specific antibody to improve specificity; membranes were then stripped (62.5 mM Tris-HCl, pH 6.8; 2% SDS; 100 mM β-mercaptoethanol, 55°C, 30 min). All protein detections were performed by standard immunoblotting protocols.

**Adipose histology and lipid analyses**

Paraffin-embedded adipose sections (5 μm) were stained with hematoxylin and eosin (H&E) and imaged by light microscopy. Adipocyte cross-sectional area was quantified using ImageJ on at least five random fields per section. TC, TG, LDL, and HDL were measured using commercial ELISA kits (Elabscience) according to manufacturer instructions. Alanine aminotransferase (ALT) and aspartate aminotransferase (AST) activities were measured using colorimetric kits (Elabscience).

**Statistical analysis**

Data are presented as mean $\pm$ standard error of the mean (SEM). Statistical comparisons were made using one-way ANOVA followed by Tukey’s post hoc test for multiple comparisons, or Student’s t-test for pairwise comparisons. P $<$ 0.05 was considered statistically significant.

**References**

Baird, R.D., Linossi, C., Middleton, M., Lord, S., Harris, A., Rodón, J., Zitt, C., Fiedler, U., Dawson, K.M., Leupin, N.*, et al.* (2021). First-in-Human Phase I Study of MP0250, a First-in-Class DARPin Drug Candidate Targeting VEGF and HGF, in Patients With Advanced Solid Tumors. Journal of clinical oncology : official journal of the American Society of Clinical Oncology *39*, 145-154.

Chuong, V., Farokhnia, M., Khom, S., Pince, C.L., Elvig, S.K., Vlkolinsky, R., Marchette, R.C., Koob, G.F., Roberto, M., Vendruscolo, L.F.*, et al.* (2023). The glucagon-like peptide-1 (GLP-1) analogue semaglutide reduces alcohol drinking and modulates central GABA neurotransmission. JCI insight *8*.

Piątkowska-Chmiel, I., Wicha-Komsta, K., Pawłowski, K., Syrytczyk, A., Kocki, T., Dudka, J., and Herbet, M. (2025). Beyond Diabetes: Semaglutide's Role in Modulating Mood Disorders through Neuroinflammation Pathways. Cellular and molecular neurobiology *45*, 22.

Rothenberger, S., Hurdiss, D.L., Walser, M., Malvezzi, F., Mayor, J., Ryter, S., Moreno, H., Liechti, N., Bosshart, A., Iss, C.*, et al.* (2022). The trispecific DARPin ensovibep inhibits diverse SARS-CoV-2 variants. Nature biotechnology *40*, 1845-1854.

Zeng, Y., Jiang, M., Robinson, S., Peng, Z., Chonira, V., Simeon, R., Tzipori, S., Zhang, J., and Chen, Z. (2022). A Multi-Specific DARPin Potently Neutralizes Shiga Toxin 2 via Simultaneous Modulation of Both Toxin Subunits. Bioengineering (Basel, Switzerland) *9*.

**Supplementary Figures**

**Figure S1**

**Fig. S1. Phage ELISA screening of DARPin clones for GLP-1R ECD. (A)** Scatter of target/blank signal ratios for individual phage-displayed DARPin clones (each dot $=$ one clone); left $=$ round 3, right $=$ round 4. **(B)** ELISA OD₄₅₀ for GLP-1R ECD (blue) versus milk control (pink) across selection rounds; Fab 3F52 shown as positive control. **(C)** Sequence alignment of G3 and G6.

**Figure S2**

**Figure S2. The predicted structure of GLP1R in complex with G3 and G6.** G3 (blue), G6 (purple), GLP-1 (red), and GLP-1R (light gray).

**Figure S3**

**Fig. S3. Competitive binding assay of G3 and G6.** Competitive binding assay in which pre-bound G3 reduced the response to subsequent G6 injection.

**Figure S4**

**Fig. S4. Residue-level binding probability of G3 derived from 815 high-QA G3-GLP-1R models.** Residue-level binding probabilities were calculated across an ensemble of 815 high-QA models (QA scores 0.758-0.794). The bar heights represent the fraction of models in which each residue was identified as part of the binding interface, with higher values indicating more consistently observed interface residues.

**Figure S5**

**Figure S5.** A diagram of the bivalent G3-G6 construct, in which the two DARPins are tethered by a (GGGGS)₂ linker.

**Figure S6**

**Fig. S6.** **Functional validation of GLP-1R signaling with semaglutide.** Dose-response of semaglutide in CRE-luc reporter CHO-GLP-1R cells.

**Figure S7**

**Fig. S7. Treatment-induced body weight trends.** Semaglutide induced sustained weight loss, G3G6 caused an initial loss followed by stabilization, while PBS control group gained weight.

**Figure S8**

**Fig. S8.** **Food intake during treatment. (A)** Record of the average food intake of mice in each group on days 6, 12, and 18 of the experimental period. **(B)** Summary table of food intake metrics for each group, including values on Day 6, Day 12, and Day 18 (g), average intake (g), total intake (g), and percentage change versus PBS (%).

**Figure S9**

**Fig. S9.** **Effects of G3G6 on body composition and adipose tissue distribution. (A)** Skeletal muscle mass (% body weight). **(B)** WAT mass (% body weight). **(C-E)** Analysis of regional adipose distribution. The proportions of **(C)** perirenal, PBS vs SE, P = 0.0095, **(D)** epididymal, PBS vs SE, P = 0.018, and **(E)** inguinal adipose depots are calculated relative to total WAT mass, PBS vs SE, P = 0.0002. Statistical significance is indicated as follows: *P < 0.05; **P < 0.01; ***P < 0.001; ****P < 0.0001; ns, not significant.

**Figure S10**

**Fig. S10. Quantification of Western blot results in brown and white adipose tissues.** UCP1, PHB1, and AKT were detected by immunoblotting, with α-tubulin and vinculin used as a loading control. (A-C) Brown adipose tissue (BAT): UCP1 (semaglutide vs PBS, P = 0.0017; G3G6 vs PBS, P = 0.0026), total AKT, and PHB1. (D-G) White adipose tissue (WAT): UCP1 (G3G6 vs PBS, P = 0.0007), PHB1 ( semaglutide vs PBS, P = 0.0331; G3G6 vs PBS, P = 0.0253), total AKT (G3G6 vs PBS, P = 0.0170), and phosphorylated AKT (pAKT) (G3G6 vs PBS, P = 0.0043). Statistical significance is indicated as follows: *P < 0.05; **P < 0.01; ***P < 0.001; ****P < 0.0001; ns, not significant.

**Figure S11**

**Fig. S11. Effects of G3G6 on lipid homeostasis in obese mice. (A-D)** Serum TC (semaglutide vs PBS, P $=$ 0.0115), TG (G3G6 vs PBS, P $=$ 0.0297), LDL (G3G6 vs PBS, P $<$ 0.0001), HDL (G3G6 vs PBS, P $=$ 0.0308), and levels after treatment. **(E, F)** Serum AST (semaglutide vs PBS, P $=$ 0.0003; G3G6 vs PBS, P $=$ 0.0294) and ALT (semaglutide vs PBS, P $=$ 0.0093) activities in PBS, semaglutide, and G3G6 groups. Statistical significance is indicated as follows: *P $<$ 0.05; **P $<$ 0.01; ***P $<$ 0.001; ****P $<$ 0.0001; ns, not significant.

**Supplementary Tables**

**Table S1**

| G3 | | | G6 | | |
| --- | --- | --- | --- | --- | --- |
| No. | Residue ID | Min Distance (Å) | No. | Residue ID | Min Distance (Å) |
| 1 | 76 | 3.333 | - | - | - |
| 2 | 77 | 3.595 | - | - | - |
| 3 | 78 | 3.673 | 1 | 78 | 3.091 |
| 4 | 79 | 3.452 | 2 | 79 | 4.680 |
| 5 | 80 | 2.783 | 3 | 80 | 3.235 |
| 6 | 82 | 3.455 | - | - | - |
| 7 | 97 | 2.795 | - | - | - |
| 8 | 98 | 4.129 | - | - | - |
| 9 | 99 | 3.262 | - | - | - |
| 10 | 101 | 2.814 | 4 | 101 | 2.478 |
| 11 | 103 | 3.607 | 5 | 103 | 2.883 |
| 12 | 111 | 3.533 | 6 | 111 | 3.359 |
| 13 | 112 | 2.991 | 7 | 112 | 4.915 |
| 14 | 113 | 3.570 | 8 | 113 | 2.414 |
| 15 | 114 | 3.460 | 9 | 114 | 3.168 |
| 16 | 120 | 2.749 | 10 | 120 | 2.554 |
| 17 | 122 | 2.826 | - | - | - |
| 18 | 124 | 2.660 | 11 | 124 | 2.656 |
| 19 | 125 | 2.382 | 12 | 125 | 2.490 |
| - | - | - | 13 | 127 | 2.563 |
| - | - | - | 14 | 130 | 4.561 |

**Tab. S1. Predicted interface residues on GLP-1R for DARPins G3 and G6.** Residues of GLP-1R predicted to interact with DARPins G3 and G6 from the top-ranked complex model are listed along with the minimum distance (Å) between any heavy atom of GLP-1R and any heavy atom of the DARPin residue.

**Table S2**

| G3 | | | G6 | | |
| --- | --- | --- | --- | --- | --- |
| No. | Residue ID | Min Distance (Å) | No. | Residue ID | Min Distance (Å) |
| 1 | 78 | 2.382 | 1 | 67 | 2.414 |
| 2 | 45 | 2.660 | 2 | 111 | 2.478 |
| 3 | 33 | 2.749 | 3 | 44 | 2.563 |
| 4 | 67 | 2.783 | 4 | 78 | 2.656 |
| 5 | 77 | 2.814 | 5 | 133 | 2.883 |
| 6 | 44 | 2.826 | 6 | 11 | 3.046 |
| 7 | 65 | 3.281 | 7 | 66 | 3.168 |
| 8 | 11 | 3.292 | 8 | 100 | 3.235 |
| 9 | 133 | 3.333 | 9 | 34 | 3.679 |
| 10 | 36 | 3.335 | 10 | 102 | 3.776 |
| 11 | 34 | 3.533 | 11 | 12 | 4.561 |
| 12 | 100 | 3.595 | 12 | 33 | 4.643 |
| 13 | 66 | 3.607 | 13 | 69 | 4.987 |
| 14 | 111 | 3.658 |  |  |  |
| 15 | 69 | 4.142 |  |  |  |
| 16 | 41 | 4.338 |  |  |  |
| 17 | 32 | 4.511 |  |  |  |
| 18 | 99 | 4.610 |  |  |  |
| 19 | 113 | 4.975 |  |  |  |

**Tab. S2. Predicted interface residues on DARPins G3 and G6 for GLP-1R.** Residues of G3 and G6 predicted to interact with GLP-1R from the top-ranked complex model are listed along with the minimum distance (Å) between any heavy atom of the DARPin and any heavy atom of GLP-1R residue.
